# Relating thalamic neuronal activity and EMG for validating predictive control of deep-brain stimulation in Essential Tremor patients

**DOI:** 10.1186/1471-2202-12-S1-P129

**Published:** 2011-07-18

**Authors:** Daniel Graupe, Ishita Basu, Daniela Tuninetti, Konstantin V Slavin

**Affiliations:** 1 Dept. of Electrical and Computer Engineering, University of Illinois, Chicago, IL, USA; 2 Dept. of Neurology and Rehabilitation, University of Illinois, Chicago, IL, USA; 3 Dept. of Neurosurgery, University of Illinois, Chicago, IL, USA

## 

Neuronal activity at the VIM nucleus of the thalamus was recorded in Essential Tremor (ET) patients during implantation of deep-brain stimulation (DBS) electrodes and compared with surface-EMG (sEMG) taken both during implantation and at later outpatient sessions at University of Illinois Hospital, Chicago. The goal of these studies was to investigate if it is possible to use sEMG signals for predicting onset of tremor and consequently whether predictive EMG-control of DBS in ET patients does or does not contradict predictive features of neuronal activity in the brain. For this purpose, we examined spike-rate and local field potentials (LFP) at and in the vicinity of VIM. Specifically, we compared spike-rate, LFP and sEMG recorded before versus after applying short DBS pulse-trains during implantation surgery, as well as sEMG recorded from limbs or neck of the patients. Out of 4 ET patients involved in the tests, we have implant-session data for 3 patients. Three patients had follow-up EMG testing.

## Results

**Figure 1:**
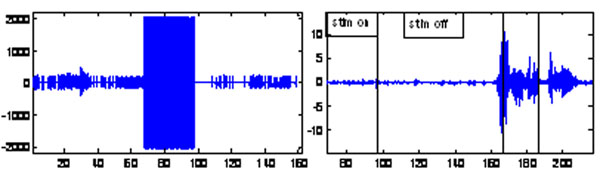
Neuronal spikes before and after stimulus (left), sEMG during and after stimulus (right)

Results show that spike rate dropped from 15.94 spikes/s to 0.97 at the end of a DBS pulse-train in patient ET1, from 45.44 to 34.78 in patient ET3 and from 22.9 to 0.26 in Patient ET 4, for periods on 12-40 seconds (Fig 1-left). LFP power dropped in one of the 2 patients where it was measured. EMG recordings are presented in [1], yielding parameters that allow prediction of onset of tremor in every cycle of DBS-on/off, noting the delayed onset of tremor. Similar results were obtained in 2 of the remaining 3 patients (also, see Fig. 1-right). Prediction of onset of tremor, at end of a train of stimuli, is based on EMG wavelets and entropy parameters derived from the signal, rather than on EMG power [1].

## Conclusions

All neuronal activity results (patients ET1, ET3, ET4) indicate sharp drops in thalamic spike rate at cessation of a simulation pulse train of 15-40 seconds as was the case in one of the two patients were LFP power was measured. The delays were all of the order of the delays (12-40 sec.) that were found in sEMG parameters at implantation sessions and at follow up session, and which we successfully used to predict onset of tremor at the cessation of a DBS-train [1]. In one patient, where one could both record EMG and visually observe the onset of tremor at end of a DBS-train, these drops agree (within 1 sec.) with changes in EMG parameters and re-appearance of tremor, to allow prediction of onset of tremor [1]. See Fig. 1. We also show that both LFP and raw sEMG allow discriminating tremor from voluntary movements (using wavelet parameters at certain bands) when DBS is OFF, as is essential for DBS control. While the results are preliminary, they point to the validity of using sEMG for control of DBS, at least in some ET patients
